# Nano-Silica Modified with Diamine for Capturing Azo Dye from Aqueous Solutions

**DOI:** 10.3390/molecules27113366

**Published:** 2022-05-24

**Authors:** Enshirah Da’na

**Affiliations:** Biomedical Engineering Department, Faculty of Engineering, King Faisal University, P.O. Box 400, Al-Ahsa 31982, Saudi Arabia; edana@kfu.edu.sa; Tel.: +966-135897540; Fax: +966-135899557

**Keywords:** adsorption, azo dye, methyl orange, nano-silica, nanocomposite, wastewater

## Abstract

Nano-silica particles decorated with amine groups (S-DA) were prepared via a simple, one-pot method, and under very mild conditions in an attempt to improve the affinity of the silica nanoparticles toward capturing anionic organic dye, namely, methyl orange (MO). The prepared sample was characterized by different techniques such as XRD for crystallinity, SEM for morphological structure, TGA for thermal stability, BET surface area, and FTIR for surface functional groups. The prepared sample was tested for the removal of MO under different conditions including the mass of adsorbent, pH, initial concentration, and time. Results showed that the adsorption of MO was very fast with equilibrium achieved in less than 30 min and a maximum removal efficiency of 100% for a mass to volume ratio of 10 g/3 L, a pH of 2.5, initial concentration of 10 mgL^−1^, and under stagnant conditions. These results were compared with a bare nano-silica, which was not able to adsorb more than 3% after 24 h, indicating the important effect of amine groups. Furthermore, recycling the adsorbent was achieved by rinsing the MO-loaded adsorbent with a dilute solution of KOH. The adsorbent maintained 50% of its initial removal efficiency after four adsorption–desorption cycles.

## 1. Introduction

Human activities such as in the textile, paint, plastic, leather, and printing industries have resulted in the discharge of a huge amount of organic dyes into the aquatic system. These organic dyes may leak and reach the groundwater, resulting in a huge spread of these highly soluble, toxic, carcinogenic, and non-biodegradable pollutants [1,2]. Furthermore, the contamination of surface water with these organic dyes negatively affects the aquatic ecosystem due to its intense color [3]. The intense color hinders the transmission of sunlight, which disturbs aquatic plants [2]. Accordingly, it is crucial to remove these dyes from wastewater before being discharged into the aquatic system. For this current research, the focus will be on methyl orange (MO), which is an anionic azo dye used for many applications such as in the textile industry and as pH indicators [4].

Many techniques have been applied for the removal of methyl orange such as precipitation [5], coagulation and flocculation [6], photodegradation [7], biological degradation [8], catalytic degradation [9,10,11], and adsorption [10,12,13,14]. Adsorption is considered among the best techniques for water treatment [15,16]. This is mainly due to its simplicity, low cost compared to other methods, low maintenance requirements, high selectivity, low chemical consumption, fast kinetic ability, and high sensitivity even at low concentration levels of the dyes [4].

Thus far, many adsorbents have been reported for the removal of methyl orange such as chitosan derivatives [1,17,18], agriculture waste [2,19,20,21], biochar [3,22], clay [23], activated carbon, mesoporous silica, alumina [24], carbon nanotubes [25], nanocomposites [10,12], polymers, and many others [4]. Despite all the progress in this area of research, many challenges still face the application of adsorption at commercial levels due to one or more of the following adsorbent drawbacks: a low adsorption capacity, slow rate of adsorption, biodegradation of the adsorbent, low stability, high cost, complex synthesis procedure, and low selectivity and sensitivity at low concentration levels. Thus, the development of new adsorbents that overcome these drawbacks is still needed. It has been reported that nanoparticles are now one of the most utilized adsorbents for this application [4].

Nano-silica has many attractive properties such as a low cost, high surface area, and being rich in silanol groups, which make it easy to introduce specific functional groups according to the target application [26,27], its nontoxic and biocompatible nature, and its high thermal and chemical stabilities. Furthermore, the structural and morphological properties of nano-silica can be tuned by controlling the synthesis conditions [11]. Amino groups have been utilized as efficient chelating groups for the adsorption removal of heavy metal ions due to their high affinity toward amines [28]. Furthermore, introducing amine affects the surface properties such as zero-charge point and hydrophilicity. Zhang et al. (2017) prepared a nano-silica-supported thiosemicarbazide–glutaraldehyde polymer via three successive steps and used the resultant nanocomposite for the adsorption of Au(III) [28]. Even though they reported a good removal efficiency and high stability, the synthesis they followed is still considered complicated since it required reflux for 18 h and heating at 90 °C [28]. Rita et al. (2018) prepared Aminopropyltrimethoxysilane-modified nano-silica via multiple steps with multiple acids, a long reflux, and heating at a high temperature of 90–110 °C for 12 h, which is also considered a complicated and costly technique [29]. Accordingly, the main goal of this work was to prepare amine-modified nano-silica following a simple route and under very mild conditions without the need for heating, reflux, or multi-steps, and applied under very simple conditions (no stirring and simple regeneration), in an attempt to reach a commercially applicable method of synthesis and treatment.

## 2. Materials and Methods

### 2.1. Chemicals

98% methanol and tetraethylorthosilicate (TEOS) were purchased from Merck (Kenilworth, NJ, USA); 99.5% cyclohexane, Triton X-100, and N-[3-(trimethoxysilyl)-propyl]ethylenediamine (DA) were purchased from Sigma-Aldrich (St. Louis, MO, USA).

### 2.2. Synthesis of Amine-Modified Nano-Silica

A microemulsion was prepared by mixing 42 g of Triton X-100 (the surfactant), 37.5 g of methanol (the co-surfactant), and 20 g of cyclohexane (the oil phase). The mixture was stirred at room temperature and 700 rpm for 30 min. After that, the pH was adjusted to a basic level by adding 4 mL of NH_4_OH to enhance the polymerization and growth of silica nanoparticles. In another flask, 5 g of TEOS was mixed with 25 mL of water for 15 min and then added to the microemulsion. After 15 min of stirring, 3 mL of DA was added dropwise and the mixture was stirred for 24 h. Then, ethanol was added to the mixture and stirred for 5 min before being filtered, washed with ethanol, and dried under ambient conditions for 24 h. The resulting nanocomposite (S-DA) was then collected and kept in a sealed bottle. For comparison purposes, the same steps were performed without adding DA to prepare nano-silica (S).

### 2.3. Adsorption of Methyl Orange Dye

An MO solution was prepared with a concentration of 50 mgL^−1^. Two sets of experiments were performed to study the effect of the mass of adsorbent, pH, and time. The first set of experiments was performed by mixing MO solution (pH = 3.5, 50 mgL^−1^) and the adsorbent with a mass to volume ratio of 10/3, 20/3, 30/3, 40/3, and 50/3 g/L. The mixture was left for 24 h under stagnant conditions. Samples were analyzed at different time intervals using UV absorbance at ʎmax of 465 nm using a UV-Vis spectrophotometer (Shimadzu, Tokyo, Japan). The same set of experiments was repeated by changing only the pH to 2.5. The removal efficiency *R*% was calculated using Equation (1)

(1) $$R\%=\frac{\;A_{i\;}-A_{t}\;}{A_{i}}\times 100$$

where *A_i_* is the initial absorbance of MO at ʎ_max_, while $A_{t}$ is the absorbance at time *t*.

For comparison purposes, the nano-silica (S) sample was also tested by mixing it with MO solution (50 mgL^−1^, 10 g/3 L) under two different pH of 2.5 and 3.5. For regeneration tests, after the S-DA adsorbent was saturated with MO, it was rinsed with a very dilute solution of KOH till the color turned from the orange back into white. Then it was dried in the oven for 1 h at 70 °C before being used for another adsorption cycle. These steps were repeated for 4 cycles.

#### 2.3.1. Adsorption Equilibrium

S-DA was tested to adsorb MO from 10–50 mgL^−1^ solution with a 10 mg mass of adsorbent. A specific mass was mixed with a specific volume of solution with a mass/volume ratio of 10/3 gL^−1^. After 24 h the S-DA sample was separated from the solution by filtration and the filtrate was analyzed by measuring the UV absorbance at ʎmax of 465 nm using a UV-Vis spectrophotometer. The adsorbed MO (*q_e_*) was calculated using Equation (2) [30,31]

(2) $$q_{e}=\frac{\left(C_{i}-C_{e}\right)V}{m}\;$$

where *C_i_* and *C_e_* are the initial and equilibrium MO concentration (mg·L^−1^), respectively. *V* is the volume of the solution in *L*, and *m* is the mass of the adsorbent in g. Langmuir adsorption isotherm was used to fit the equilibrium data using Equation (3)

(3) $$q_{e}=\frac{q_{m}K_{L}C_{e}}{1+K_{L}C_{e}}$$

where *q_m_* and *K_L_* are the equilibrium adsorption capacity (mg·g^−1^) and Langmuir constant (L·mg^−1^), respectively.

#### 2.3.2. Adsorption Kinetics

S-DA was tested to adsorb MO from a 50 mgL^−1^ solution with a different mass of adsorbent in the range of 10–50 mg. For each test, the S-DA was added to 3 mL of the solution in a UV quartz cuvette. The concentration of the MO was then followed by UV absorbance at ʎ_max_ of 465 nm using a UV-Vis spectrophotometer for 2 h. Pseudo-second-order model was used to fit the kinetic data using Equation (4) [30,31]

(4) $$q_{t}=\frac{K_{P2}q_{e}^{2}t}{1+K_{P2}q_{e}t}$$

where *q_e_* and *K_P_*_2_ are the equilibrium adsorption capacity (mg·g^−1^) and Pseudo-second-order rate constant (g·mg^−1^·min^−1^), respectively.

### 2.4. Characterization

The crystallinity of the samples was tested via X-ray diffraction using XRD-7000 with a Cu detector (Shimadzu, Tokyo, Japan). Scanning Electron Microscopy (SEM) was performed to explore the morphology of the samples using Thermo Scientific, Quattro S, USA. Furthermore, energy dispersive X-ray spectrometry (EDX) (JEOL JEM-1011-instrument) (Tokyo, Japan) was performed. Thermal gravimetric analysis (TGA) was performed to test the thermal stability of the samples using TGA-51 Shimadzu Thermogravimetric Analyzers (Tokyo, Japan) by heating the samples under the flow of nitrogen at a ramp of 2 °C/min from 25 °C to 600 °C. The surface area of the sample was estimated by nitrogen adsorption at 77 K using A NOVA 4200e (Quantachrome Instruments, B Beach, FL, USA). The specific surface area and pore size were further analyzed using the Brunauer–Emmett–Teller (BET) equation. The pore size distribution was estimated using the isothermic adsorption branch (BJH) Barrett–Joyner–Halenda technique. The functional groups on the surface of the samples were investigated via FTIR spectrometer (IR–Tracer 100 Fourier Transform Infrared Spectrophotom, Shimadzu, Japan). Dynamic light scattering (DLS) was performed using Cilas’ dual scattering particle size analyzer Nano DS (Orléans, France). A specific amount of S-DA was sonicated in a specific amount of deionized double-distilled water for 30 min with power sonic 405. The pH_ZPC_ was measured by the equilibrium technique as reported elsewhere [32]. Kjeldahl analytical titration method was used to estimate the nitrogen content of the S-DA sample [33].

## 3. Results and Discussion

### 3.1. Characterization of the Adsorbent

**Figure 1:**
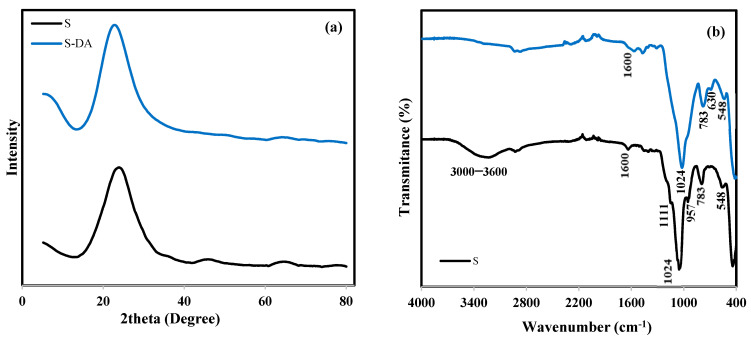

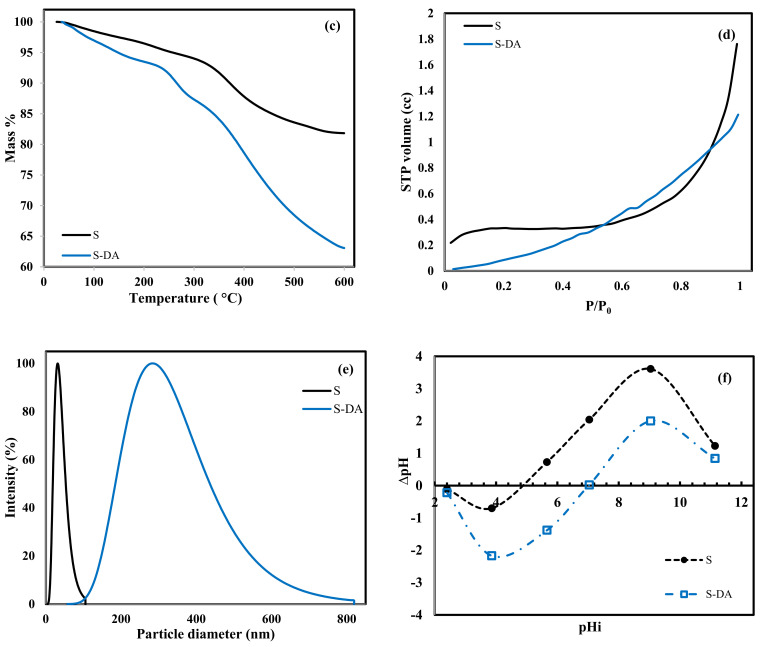
Characterization of S and S-DA by XRD (**a**), FTIR (**b**), TGA (**c**), BET analysis of surface area by nitrogen adsorption (**d**), the DLS analysis (**e**), and the pH_ZPC_ for nano-silica (S) and amine-modified nano-silica (S-DA) obtained by equilibrium technique [13] (**f**).

To examine the crystallinity of the bare silica (S) and the amine-modified silica (S-DA), the XRD spectrum was collected over the 2θ range of 5–80° at λ = 1.54056 nm and a scanning rate of 2°/s. Figure 1a shows a single broad peak at 23°, which is the characteristic of amorphous silica [34].

Figure 1b shows the FTIR spectrum of S and S-DA in the wavenumber range of 400–4000 cm^−1^. The broad peak around 3400 cm^−1^ in the S sample is related to the hydrogen bond resulting from hydroxyl (-OH) and (H_2_O) adsorbed on the surface [35]. This peak disappeared in S-DA spectra, which is mainly due to the involvement of the OH groups in the reaction with the DA. The IR band at 1111 cm^−1^ with a shoulder at 1188 cm^−1^ in the S sample is usually assigned to the Si-O-Si asymmetric stretching vibrations. The band at 1600 cm^−1^ is related to the O-H stretching and bending vibration of the adsorbed water and silanol groups [28]. The peaks around 783 and 1024 cm^−1^ are related to the stretching of Si-O and Si-O-Si bonds, respectively [36]. The weak shoulder around 957 cm^−1^ in the S sample corresponds to the Si-OH stretching vibration [36]. The peaks around 548 cm^−1^ and 783 cm^−1^ are attributed to the Si-O-Si stretching vibration and bending vibrations, respectively [36]. The weak band at around 630 cm^−1^ in the S-DA corresponds to N-H bending [36]. From FTIR analysis, it is possible to say that DA was successfully attached to the silica surface.

**Figure 2:**
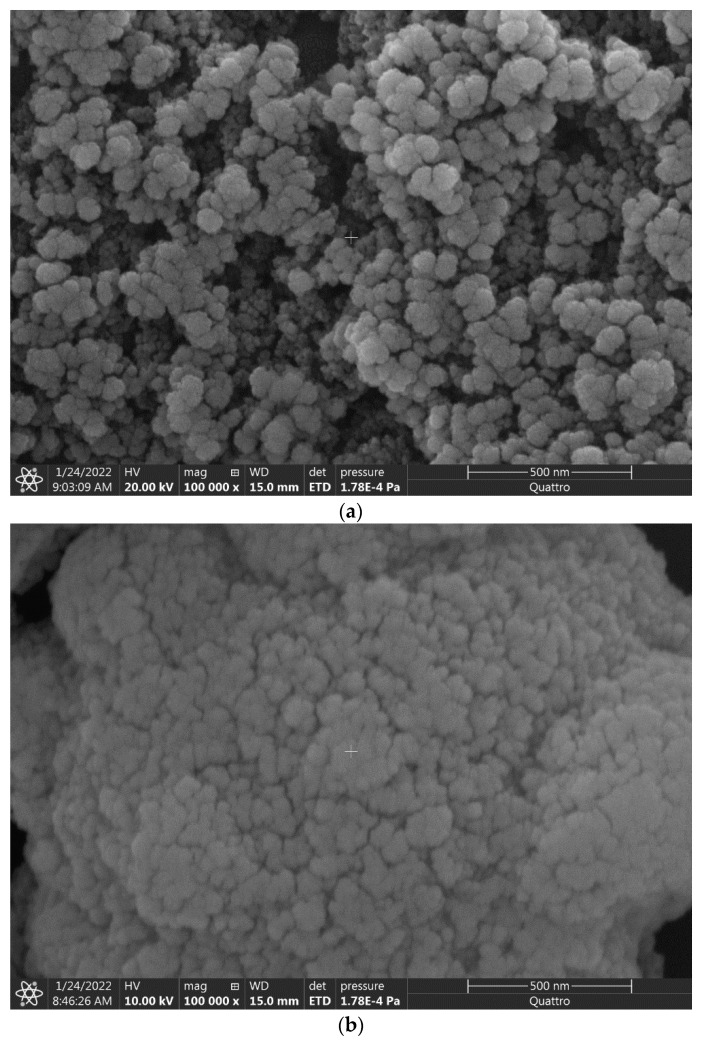

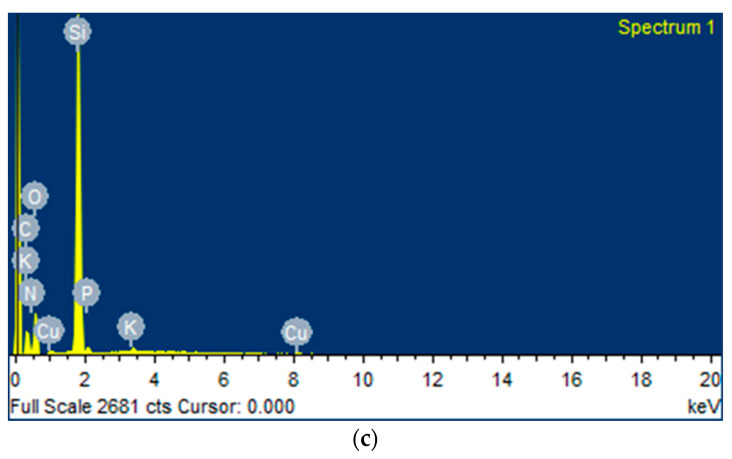
SEM images for S (**a**) and S-DA (**b**) at a magnification of 100,000×, and EDS of S-DA (**c**).

To further confirm that the DA was attached to the silica surface in S-DA, the Kjeldahl analytical titration method was used. This test showed that the sample S-DA contains 13.2% of nitrogen. In addition, the EDX was performed for the sample and the results are shown in Figure 2c. The analysis showed that the S-DA sample contains 6.12 weight % of nitrogen.

To assess the thermal stability of the adsorbent, TGA was performed in the temperature range of 20–600 °C as shown in Figure 1c. The TGA profile shows two different regions of loss. According to the literature, the first loss (≈7%) occurs in the temperature range of 20–235 °C, which is mainly related to the loss of moisture content within the sample and some other gases that may be adsorbed on the surface due to improper storage such as CO_2_ [37]. The other region extends in the temperature range of 235–600 °C and resulted in a 30% loss of the sample. This loss is mainly related to the dissociation of amino groups attached to the surface [37]. The mass loss of bare silica nanoparticles is 18%, while the total loss for S-DA is 37%. The extra 19% loss confirms the attachment of amino groups to the surface.

Figure 1d shows the nitrogen adsorption–desorption isotherm of the S and S-DA. It shows the adsorbed nitrogen at STP for P/P_0_ in the range 0–1, with P being the adsorption pressure and P_0_ being the saturation pressure at which the maximum adsorption can be achieved. The N_2_ adsorption profile shows a porous structure of the bare silica (S), which has been significantly affected after attaching amino groups in S-DA. The S isotherm indicates a mesoporous structure with a surface area of 25 m^2^g^−1^ and pore volume of 0.182 cm^3^g^−1^. Attaching DA to the silica surface resulted in a drastic change in the porous structure with a huge decrease in the specific surface area to 10 m^2^g^−1^ and the pore volume to 0.0768 cm^3^g^−1^. This decrease in the surface area and pore volume of S-DA compared to S is a result of the enhanced growth rate of the silica nanoparticles of S-DA under the effect of amine catalysis leading to the loss of the surface area. This is supported by the SEM imaging results and the DLS analysis to be discussed next.

The morphology of the prepared adsorbent was investigated by SEM imaging as shown in Figure 2. For the S sample, Figure 2a shows a mixture of spherical particles with a narrow particle size distribution and a high tendency for aggregation. However, the structure still shows some porosity within the particles. For the S-DA sample, Figure 2b shows a drastic change in the morphological structure with very low porosity. This is the reason for having a relatively low surface area and pore volume via nitrogen adsorption analysis. The dynamic light scattering result shown in Figure 1e also confirms the difference between the two samples with a very narrow particle size distribution of S around 35 nm and wide distribution of particle size for S-DA in the range of 200–800 nm. This could be related to the tendency of N-[3-(Trimethoxysilyl)-propyl]ethylenediamine to condensate and block the porosity within the silica particles. Moreover, amine may enhance the growth rate of the silica nanoparticles leading to a larger particle size with lower porosity. Kesmez et al. (2010) reported similar findings for the effect of amine on the silica growth rate and particle size distribution. They reported silica with an average particle size around 21 nm with a narrow particle size distribution without adding amine. On the other hand, the silica obtained with the aid of amine as a catalyst showed two peaks for the particle size distribution around 44 and 500 nm [38].

### 3.2. Adsorption of MO

**Scheme 1:**
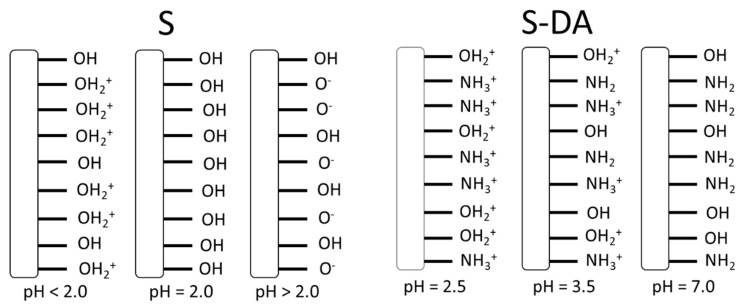
Proposed surface for nano-silica (S) and amine-modified nano-silica (S-DA).

**Figure 3:**
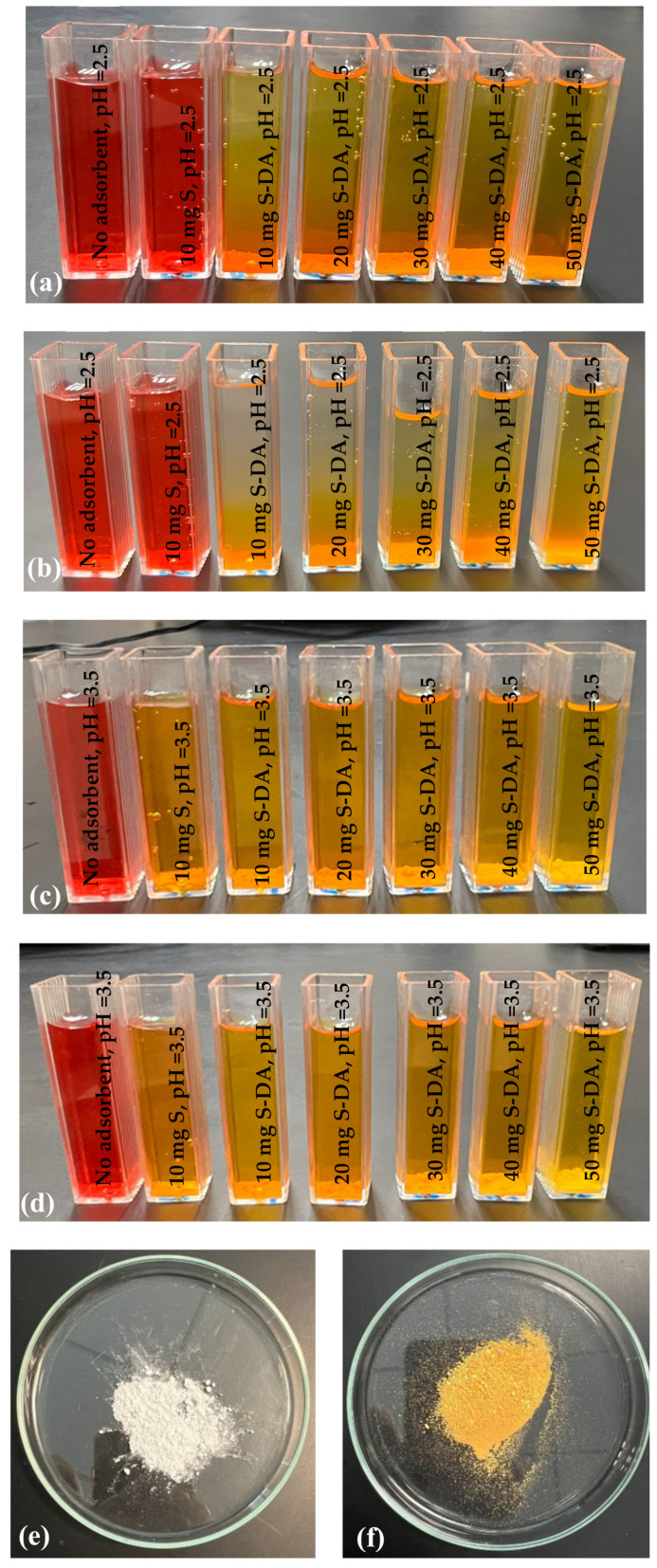
Color change by using an adsorbent to solution ratio of 10 g/3 L *C*_0_ = 50 mgL^−1^, and pH = 2.5, directly after adding S-DA (**a**) and after 2 h (**b**), pH = 3.5 directly after adding the S-DA (**c**), and after 2 h (**d**). From left to right for (**a**–**d**) without adsorbent, 10S, 10S-DA, 20S-DA, 30S-DA, 40S-DA, and 50S-DA, S-DA before being used for adsorption (**e**), and MO-loaded S-DA (**f**).

The main rule for adding amine functionality to the silica was to increase the pH_ZPC_ of the silica from 2.0 for bare silica to around 7.0 for amine-modified silica nanoparticles as shown in Figure 1f [39]. This has a major impact on the efficiency of the nanocomposite since the surface will be positively charged for any pH value less than 7. Thus, more MO anions will be attracted to the surface. A similar effect of pH on the adsorption of MO on the surface of protonated chitosan was reported by Huang et al. (2013) [17]. Lu et al. (2018) reported a decrease in the adsorption capacity of MO on the surface of biochar at a pH higher than 7 due to the deprotonation of the surface and, thus, repulsive force with the anionic dye [22]. Da’na et al. (2022) applied the amine-modified silica with a pH_ZPC_ around 7 to adsorb Zn^2+^ by controlling the solution pH at 7.5, which makes the adsorbent surface negatively charged, and they reported 100% removal of this cation [32]. Khalaf et al. (2019) applied ZnO nanoparticles to adsorb anionicbytionic dyes by controlling the surface charge of the adsorbent [13]. In this work, since the pH_ZPC_ of the S-DA adsorbent was found to be around 7.0 (Figure 1f), it is expected that the surface of S-DA will be positively charged in a medium with pH less than 7 as shown in Scheme 1. Accordingly, two sets of experiments were performed at a pH of 2.5 and 3.5. Qualitatively, Figure 3a–d show this pH effect. When the adsorbent (S-DA) is added to an MO solution at pH = 2.5, the surface of the adsorbent will be highly positive as proposed in Scheme 1, and, accordingly, there will be an electrical attraction between the positive surface and the negative MO. This is shown clearly in Figure 3a,b. The diffusion of the MO from the top of the cuvette to the bottom is very clear. The first row represents the time just after adding the adsorbent, while row 2 represents 30 min later. This Is not clear in Figure 3c,d, which represent experiments at pH 3.5. Increasing the pH is expected to decrease the density of the positive charge on the adsorbent surface (Scheme 1). Furthermore, the concentration of OH^−1^ is expected to be higher at pH = 3.5 than that at pH = 2.5. The OH^−1^ will compete with the MO anions for the positive sites on the adsorbent surface. Accordingly, slower diffusion is expected to take place at pH = 3.5 with lower removal efficiency. To confirm the rule of amine in improving the surface charge of the adsorbent, other experiments were performed with silica nanoparticles (S), which have a pH_ZPC_ of 2.0 as shown in Figure 1f [32]. Thus, at both pH values, the surface will be negatively charged as proposed in Scheme 1 and a repulsive force is expected to prevent the reach of the MO molecules to the adsorption sites on the surface. Figure 3 shows the S-DA before (Figure 3e) and after the adsorption of MO (Figure 3f). The color change was very fast for pH = 2.5 and much slower at pH = 3.5, while no change in color was detected when S was used as the adsorbent.

**Figure 4:**
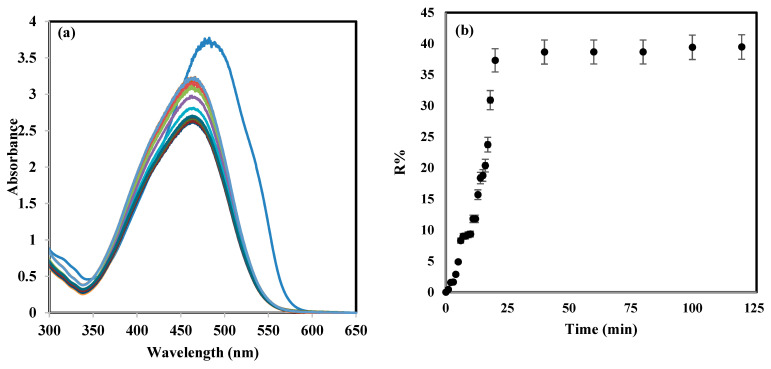
UV absorbance at ʎ_max_ = 465 nm each 3 min for 30 min (**a**), the removal efficiency (*R*%) by using an adsorbent to solution ratio of 10 g/3 L, *C*_0_ = 50 mgL^−1^, and pH = 2.5 for 2 h (**b**).

**Figure 5:**
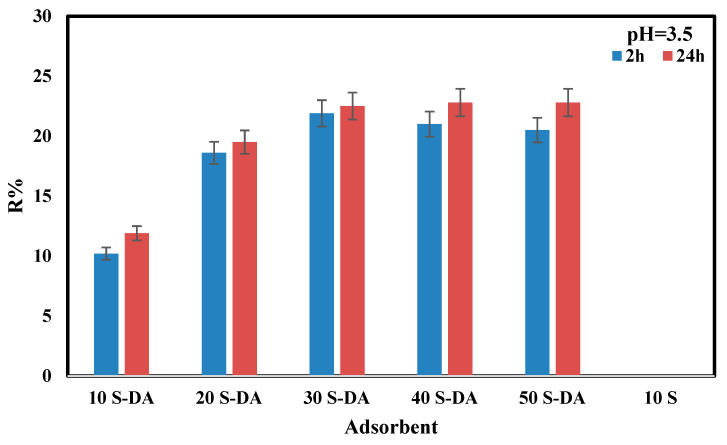
The removal efficiency (*R*%) was reported for S (10 g/3 L) and S-DA with *C*_0_ = 50 mgL^−1^, mass/volume of 10 g/3 L–50 g/3 L, pH = 3.5, for 2 and 24 h.

**Figure 6:**
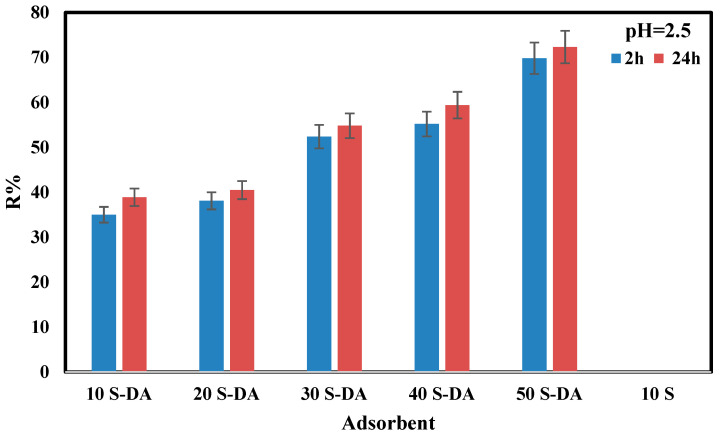
The removal efficiency (*R*%) was reported for S (10 g/3 L) and S-DA with *C*_0_ = 50 mgL^−1^, mass/volume of 10 g/3 L–50 g/3 L, pH = 2.5, for 2 and 24 h.

To quantitatively estimate the efficiency of adsorption, the UV absorbance at ʎ_max_ of 465 nm using a UV-Vis spectrophotometer was followed for the time of the experiment as shown in Figure 4a. Each spectrum represents the absorbance at a certain time, starting from the top at time zero and ending at the lowest one by the end of the experiment, with a 3 min interval between each. Figure 4b shows the kinetic data of the removal efficiency for one experiment using 10 g/3 L of S-DA at pH = 2.5. The kinetic energy was relatively fast with the equilibrium reached in less than 30 min. Similar experiments were repeated for different adsorbent to solution ratios of 20 g/3 L, 30 g/3 L, 40 g/3 L, 50 g/3 L, and a pH of 3.5, and the results are presented in Figure 5 and Figure 6.

Figure 5 shows the removal efficiency obtained for different mass/volume ratios, with a pH of 3.5, for 2 h and 24 h. The maximum removal efficiency ≈ of 22% was achieved with 30 mg/3 L after 2 h. For bare silica nanoparticles (S) the removal efficiency was 0%, which is mainly due to the low pH_ZPC_ of the silica. The pH_ZPC_ of 2 implies that the surface of the silica will be negatively charged at any pH higher than 2 as proposed in Scheme 1. Thus, a repulsive force between the negative silica surface and the MO anions will prevent the reach to the active sites and, accordingly, no adsorption will take place. Decreasing the pH to 2.5 resulted in a higher removal efficiency of 70% as shown in Figure 6. The enhancement of the removal efficiency is directly related to the charge of the surface. By decreasing the pH, more protonation of the functional groups on the surface will take place leading to a higher density of positive charge on the surface as proposed in Scheme 1, which will attract more of the MO anions. Bare silica also did not adsorb any MO at this pH.

**Figure 7:**
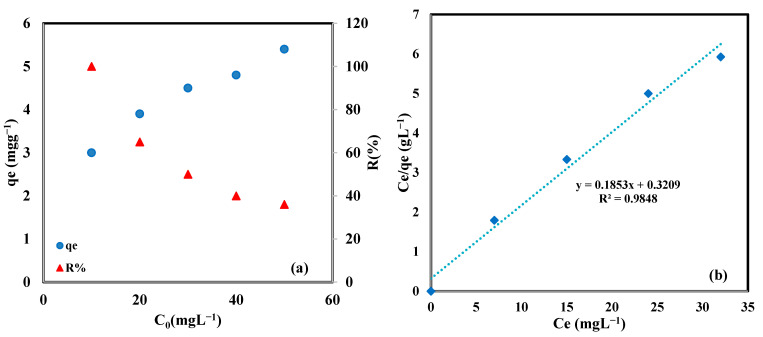

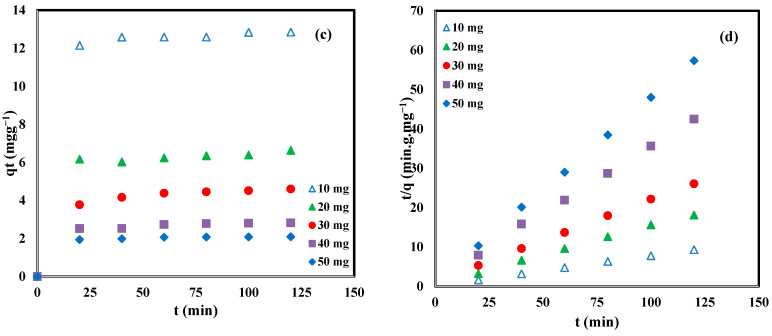
Adsorption capacity (*q_e_*) and removal efficiency (*R*%) as a function of *C*_0_ (**a**), Langmuir model (**b**), adsorption capacity (*q_t_*) as a function of time (**c**), and kinetics model (**d**) for S-DA with mass/volume of 10 g/3 L–50 g/3 L, pH = 2.5, for 2.

To further assess the adsorption process, the equilibrium was investigated by adsorbing MO from solutions with different initial concentrations in the range of 10–50 mgL^−1^ and 293 K. Figure 7a shows that a 100% removal was achieved when a dilute solution of 10 mgL^−1^ was used. The result was fitted with the Langmuir isotherm as shown in Figure 7b suggesting monolayer adsorption. This supports the suggested mechanism of electrical attraction between the surface and the MO, which required direct contact between the positive surface and the anions. Thus, it will take place in a single layer. Table 1 shows the fitting parameters with an R^2^ of 0.9848 and a monolayer adsorption capacity of 5.4 mg/g. The same test was repeated at 313 and 333 K to investigate the thermodynamics of the process. When the temperature increased, no adsorption was achieved, which indicates an exothermic nature of the process. As mentioned earlier, the main expected mechanism of the adsorption is via electric attraction between the MO anions and the positive S-DA surface. By increasing the temperature, the MO molecules gain kinetic energy that can overcome the electrical attraction forces and, accordingly, no adsorption is achieved. The kinetics of the adsorption process were investigated at 293 K using an initial concentration of 50 mgL^−1^, pH of 2.5, and different mass of adsorbent in the range of 10–50 mg as shown in Figure 7c. It is clear that for all kinetic tests, the adsorption was very fast and the equilibrium was achieved in less than 30 min. The kinetic data were fitted with the pseudo-second-order model as shown in Figure 7b and Table 1. The maximum adsorption capacity was achieved with 10 mg of adsorbent and decreased as the adsorbent mass increased. For the rate constant *K_P_*_2_, its value increased by increasing the mass from 10 to 30 mg and then started to decrease.

Table 2 shows some adsorbents reported in the literature for the removal of MO from aqueous solutions. It is worth mentioning that it is not easy to compare different adsorbents and draw a comprehensive conclusion about which one is the best. This is mainly due to the different experimental conditions followed in each work such as temperature, pH, concentration range, mass/volume used, and many other conditions. In addition to these experimental conditions, many other factors need to be considered such as the stability and reusability of the adsorbent, simplicity of synthesis, and cost. It is apparent in the table that many adsorbents (naturally occurring materials such as agriculture waste) show very high adsorption capacity and are also available at low or no cost. However, these adsorbents are usually unstable and cannot be used for multiple cycles, and there needs to be a suitable way of disposing of them to prevent the leak of the pollutants back into the water body. To find a conclusion, a comprehensive study of each adsorbent must be conducted to achieve an adsorbent that can be commercially applied.

### 3.3. Regeneration of the Adsorbent

**Figure 8:**
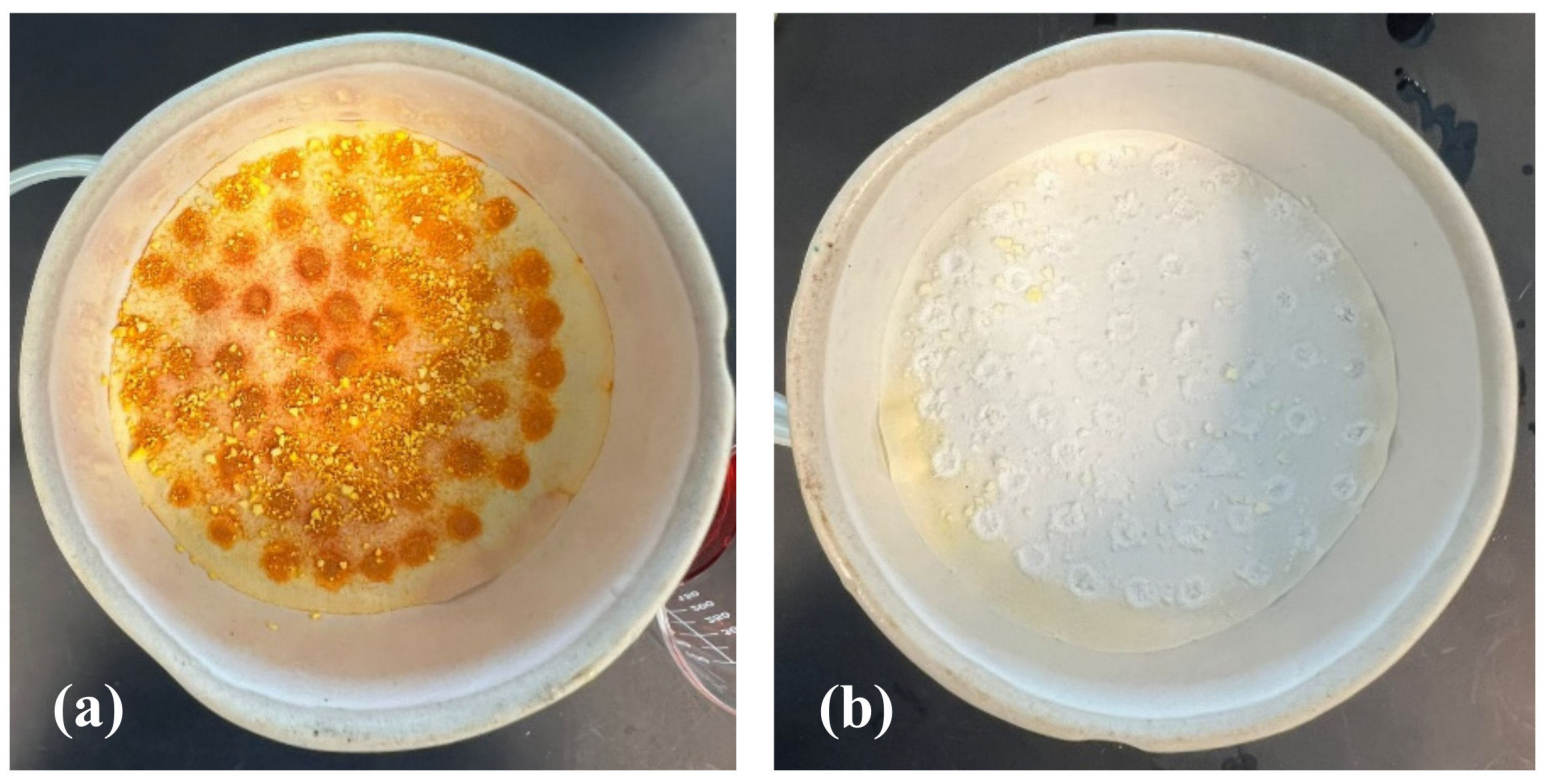
MO-loaded S-DA (**a**) and S-DA after releasing MO by KOH (**b**).

**Figure 9:**
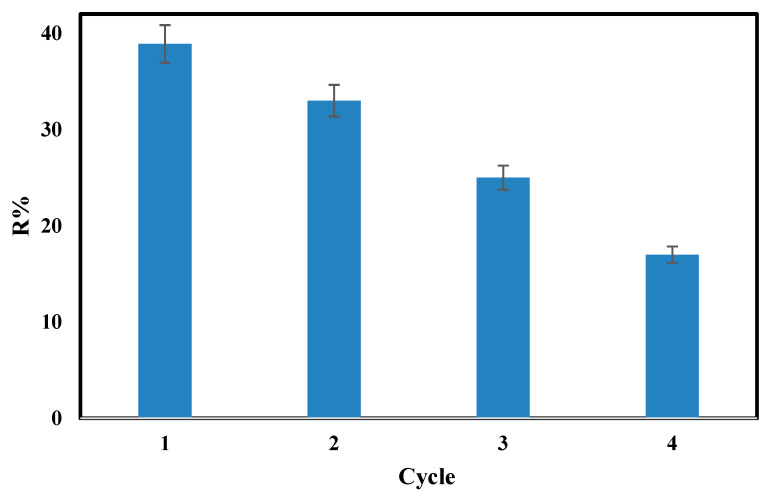
The removal efficiency was obtained by 10 g/3 L of S-DA, pH = 2.5, for 2, recycling with KOH four times.

**Scheme 2:**
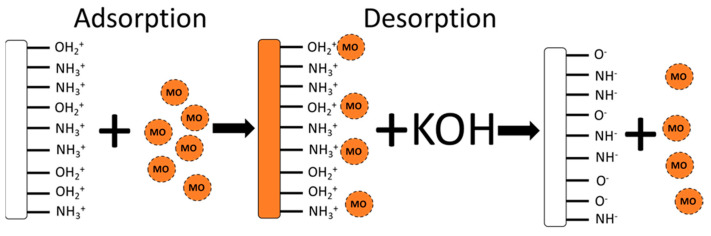
Proposed mechanism of the adsorption–desorption cycle of methyl orange on the surface of amine-modified nano-silica (S-DA).

The regeneration test was performed with a 10 g/3 L ratio for four successive adsorption–desorption cycles by rinsing the amine-loaded S-DA shown in Figure 8a with a diluted KOH solution to change the surface charge from positive to negative as proposed in Scheme 2. Thus, a repulsive force results in the release of the MO anions into the KOH solution. Visually, it is apparent that MO was successfully released from the adsorbent surface by retaining the white color of S-DA as shown in Figure 8b. This step was performed by adding KOH with a dropper directly on the filter paper for less than 5 min. The result of reusing the adsorbent for four successive cycles is shown in Figure 9. The S-DA adsorbent maintained 50% of its initial removal efficiency after being used for four successive adsorption–desorption cycles. The loss of adsorption capacity may be related to the incomplete release of the MO from the surface due to the very short contact time. It is possible also that the successive acid-base contact with the S-DA resulted in the loss of amine functionality and, accordingly, the surface chemistry was affected. Moreover, it is possible that the KOH treatment caused some changes in the morphological structure of the adsorbent surface due to the itching effect.

## 4. Conclusions

In this work, amine-modified nano-silica was prepared following a simple, one-pot method, and without the need for any harsh conditions such as a strong acidic or basic medium, high temperature, or reflux. The main rule of introducing amine was to enhance the affinity of the silica surface to capture the MO dye by increasing the zero-point charge of the silica surface from 2.0 for bare silica to about 7.0 for the S-DA. The prepared adsorbent showed fast kinetics toward capturing MO with equilibrium achieved in less than 30 min under stagnant conditions. Regeneration of the adsorbent was performed by simple rinsing of the MO-loaded adsorbent with a dilute KOH solution. After four cycles the adsorbent maintained 50% of its initial adsorption capacity. This material is very attractive for this application; however, more research is needed to improve the adsorption capacity of the adsorbent by optimizing the synthesis conditions followed in this work.

## Figures and Tables

**Table 1:** Equilibrium and kinetics parameters were obtained for S-DA with an initial MO concentration of 50 mgL^−1^, pH = 2.5, the temperature of 293 K, and mass/volume of 10/3–50/3 g/L.

Temperature (K)	Langmuir parameters		
	*K_L_* (L·mg^−1^)	*q_m_* (mg·g^−1^)	R^2^
293	0.577	5.40	0.9848
313	-	-	-
333	-	-	-
Mass (mg)	Pseudo-second-order parameters		
	*K_p_*_2_ (g·mg^−1^·min^−1^)	*q_e_* (mg·g^−1^)	R^2^
10	0.048	12.987	0.9998
20	0.041	6.716	0.9982
30	0.035	4.801	0.9998
40	0.078	2.927	0.9992
50	0.210	2.132	0.9999

**Table 2:** Some adsorbents are reported in the literature for the adsorption removal of MO.

Adsorbent	Mass/Volume (g/L)	MO Concentration (mgL^−1^)	Removal Efficiency (%)	Adsorption Capacity (mg/g)	Notes	Ref.
Chitosan intercalated montmorillonite	1/1	200		100	pH = 2 T = 45 °C	[18]
Protonated cross-linked chitosan		100	100		pH = 4.5 T = 40 °C	[17]
Sheep Manure Biochar	4/5	20	100	50	pH = 4 T = 25 °C	[22]
Kaolinite Clay	50/1	200	70	3.5	pH = 4 T = 25 °C	[23]
Camel Thorn Plant	1/2	20	80	21	pH = 4 T = 20 °C	[21]
Biochar	1/2	50		137	T = 25 °C pH = 2	[3]
CTAB-Modified Orange Peel	4/5	50	89	14	T = 25 °C	[20]
Chitosan	2/1	33		10	pH = 4 T = 33 °C	[1]
Polyvinylidene fluoride-PEDOT mats		300		293	pH = 3 T = 50 °C	[40]
Crosslinking chitosan microspheres	4/10	30	91	50	T = 25 °C	[41]
Kapok paper-zinc oxide-polyaniline hybrid nanocomposite	2/5	25	50	97	T = 25 °C	[42]
Mesoporous maghemite	5/2	50	93	385	pH = 3 T = 20 °C	[43]
Hydrogel Composite	1/1	1000		500	pH = 7 T = 15 °C	[44]
Aluminum-based MOF/graphite oxide composite	0.2/1	40		399	T = 25 °C pH = 8	[45]
Magnetic mesoporous carbon	2/1	30		98.5	pH = 7 T = 25 °C	[46]
Ferromagnetic Fe/Ni Nanoparticles	1.5/1	50	35	99.5	pH = 1 T = 25 °C	[47]
TiO_2_ nanocrystals	1/50	16	95	303	pH = 3 T = 25 °C	[48]
ZnO–NiO nanocomposite	2/1	6	3	100	pH = 4 T = 20 °C	[13]
Amine-modified nano-silica	10/3	10	5.4	100	pH = 3.5 T = 20 °C	Current work

## Data Availability

Not applicable.
